# Changing trends in a decade of vascular radiology—the impact of technical developments of non-invasive techniques on vascular imaging

**DOI:** 10.1007/s13244-012-0188-6

**Published:** 2012-08-06

**Authors:** Gordon W. Cowell, Allan W. Reid, Giles H. Roditi

**Affiliations:** Department of Radiology, Glasgow Royal Infirmary, Alexandra Parade, Glasgow, G4 0SF, Scotland United Kingdom

**Keywords:** Contrast-enhanced magnetic resonance angiography (CE-MRA), Contrast-enhanced computed tomography angiography (CE-CTA), Vascular imaging

## Abstract

**Objectives:**

This review aims to establish the impact on conventional angiography and endovascular intervention of contrast-enhanced magnetic resonance angiography (CE-MRA) and contrast-enhanced computed tomography angiography (CE-CTA) on a background of evolving technology, changing clinical requirements and resulting clinical repercussions.

**Methods:**

The angiographic and interventional caseload was prospectively recorded between 1997 and 2010, along with the CE-MRA and CE-CTA caseload. Waiting times and the marginal cost analyses for 2001 and 2009 were also prospectively established.

**Results:**

Conventional diagnostic angiographies declined from a peak of 847 to 121 per year while endovascular interventions continue in similar numbers. CE-MRA increased from effectively none initially to 620 per year while CE-CTA has currently risen to 396 per year. Total diagnostic study numbers have increased but at reduced cost. Various influences are clear, including on-site modality availability, capability and accuracy along with impact of new therapies, research studies and adverse events.

**Conclusions:**

Vascular imaging has undergone a metamorphosis in little over a decade because of CE-MRA and CE-CTA. With waiting times significantly reduced since the start of the study and the cost-effectiveness of both CE-MRA and CE-CTA as primary diagnostic investigations established, further development of these services is inevitable.

**Main Messages:**

• *The availability of CE-MRA and CE-CTA has reduced the need for conventional angiography.*

• *Both waiting times and the marginal cost analyses for CE-MRA and CE-CTA have reduced.*

• *The impact of new therapies, research studies (e.g. ASTRAL) and adverse events is illustrated.*

## Introduction

The imaging modalities of choice in vascular imaging have changed considerably over the last decade, with advances in magnetic resonance imaging (MRI) and computed tomography (CT) technology coupled with the increasing availability of MRI and multislice CT scanners capable of these forms of non-invasive angiography. Our aim in this work has been to establish the impact of the evolving technologies of conventional angiography, interventional angiography, contrast-enhanced MRI angiography (CE-MRA) and contrast-enhanced CT angiography (CE-CTA) on the respective workloads of conventional invasive angiography and vascular interventional procedures. We have also related this to the clinical requirements with emerging clinical repercussions in a referral centre along with the impact on clinical practice.

## CE - MRA

CE-MRA with gadolinium-based contrast agents has emerged as a realistic non-invasive alternative to traditional invasive diagnostic angiography. As well as the development of contrast-enhanced techniques, the ancillary technological advances in MRI software and hardware (gradient coils and phased array coils) plus bolus detection methods, stepping table techniques and parallel imaging have shortened examination times and improved image quality significantly. The use of MRI fulfils the ALARA principle (‘as low as reasonably achievable’) as regards minimising exposure to ionising radiation but there is also a favourable side effect profile in respect of the contrast agents used with extremely few patients experiencing adverse events [1, 2], with many of these just mild side effects such as nausea, vomiting or urticaria [3]. Only a limited number of reports of contrast-induced toxicity are available, with the majority of studies showing no evidence of deterioration in renal function [4]. Recently an association between the use of certain linear chelate gadolinium-based contrast agents (GBCAs) in patients with severe renal impairment (mainly those requiring dialysis) and the potential development of the rare, recently recognised condition nephrogenic systemic fibrosis (NSF) has been reported [5], leading to these particular linear chelate agents being contra-indicated in acute kidney injury and severe chronic renal failure. Cyclic gadolinium chelates have not been specifically contra-indicated.

**Fig. 1:**
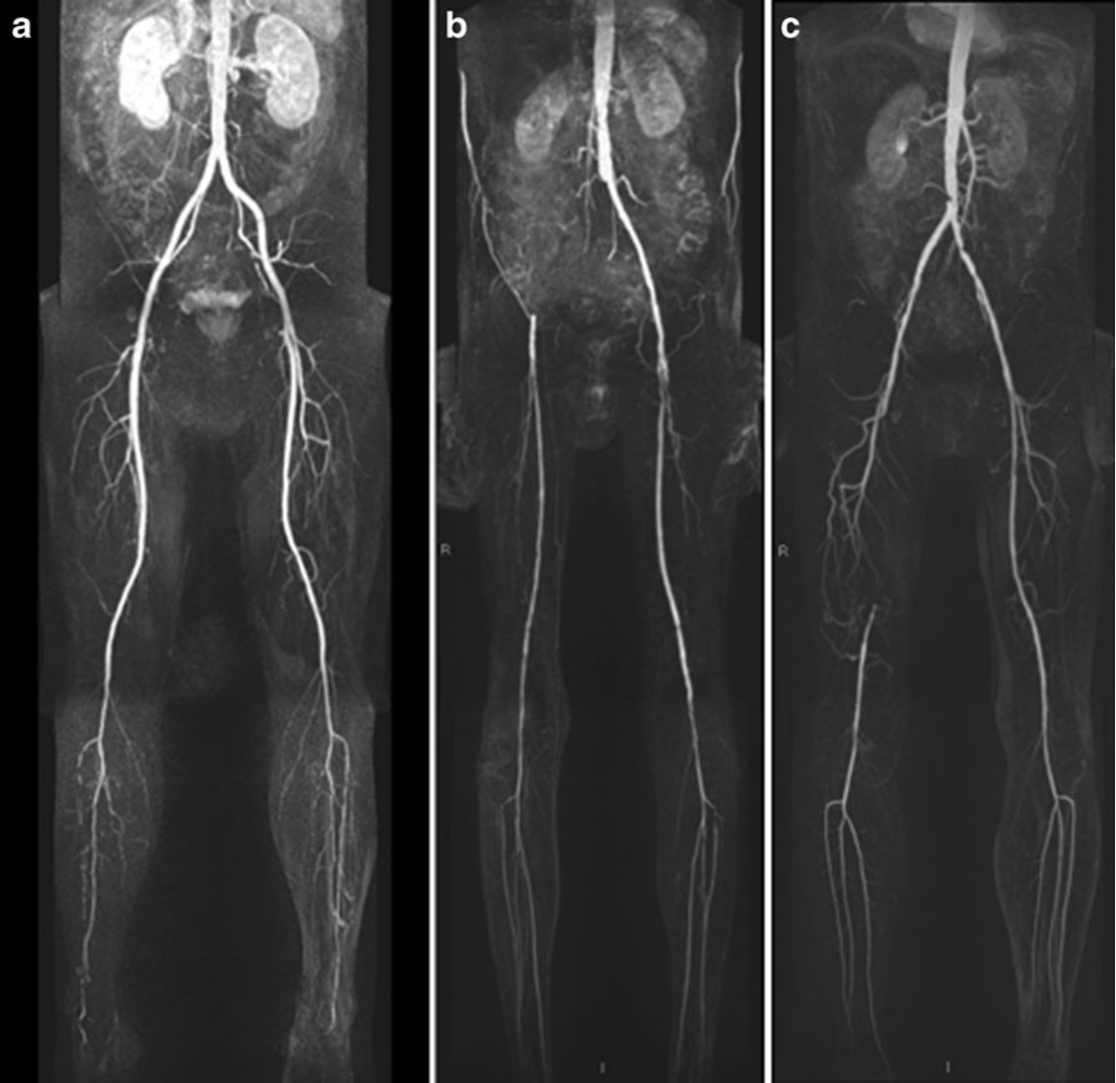
Three-station ‘bolus-chase’ moving-table lower limb MR angiograms. Left study (**a**) in a diabetic patient with calf claudication shows preserved aortoiliac and femoropopliteal segments but typical occlusive below-knee tibial disease. Middle study (**b**) in bilateral claudicant with absent right femoral pulse shows occluded right common and external iliac arteries with reasonably preserved infra-inguinal run-off on that side, whereas on the left while the iliac arteries are reasonable there is stenotic femoral artery disease in the proximal thigh. Right study (**c**) in a claudicant patient with impaired left femoral pulse reveals shelf-like lower aortic stenosis, stenotic left iliac disease and an occluded right superficial femoral artery but preserved infrapopliteal run-off. Note that in these non-critical claudicant patients there is little ‘venous contamination’, and these MRA images are as presented immediately after acquisition with automated MIP post-processing and stitching to give an instant overview vascular map of multilevel disease

Investigations with surgical/angiographic correlation have shown CE-MRA to have a high sensitivity and specificity for detection of peripheral arterial steno-occlusive disease, and 100 % sensitivity and specificity for detection of aortic or iliac aneurysms [6]. CE-MRA has hence become accepted for non-invasive angiographic studies that can be performed on an out-patient basis, with high accuracy, for example in evaluation of the peripheral (lower limb) arterial system [7]. There are some limitations, which may curb the use of MRI, including cardiac pacemakers and resynchronisation devices, ferromagnetic intracranial aneurysm clips, shrapnel injuries, claustrophobia and steel intravascular stents. The largest increase in workload was anticipated in patients undergoing CE-MRA of the abdominal aorta, iliac vessels and lower limb run-off. This is because traditionally arteriography of the aortoiliac and lower limb run-off assessing patients with peripheral arterial occlusive disease (PAOD) has contributed the largest proportion of conventional diagnostic angiography workload. We anticipated that this area would show a clear benefit from the transfer of primary investigation to a predominantly outpatient non-invasive test such as CE-MRA. An example of the use of CE-MRA for assessment of PAOD in our institution is shown in Fig. 1.

## CE-CTA

CE-CTA has also advanced rapidly in the last decade with the advent of multislice scanners with increasing numbers of detector rows, accompanied by bolus detection methods and increasingly rapid rotation of CT gantries that have allowed increased spatial resolution and extended coverage. These technological advances have improved CE-CTA accuracy, and 64-detector row CE-CTA has been shown to be superior to 4- or 16-detector row studies demonstrating high sensitivity and specificity, in comparison with digital subtraction angiography [8]. CT has some advantages compared to MRI in that patient features such as pacemakers pose no problem and intravascular stents including those made of steel are well assessed. However, CT angiographic examinations are high-radiation-dose studies limiting their repeated use in younger patients while iodine-based contrast media carry a risk in patients with renal impairment lest contrast-induced nephrotoxicity (CIN) further damages kidney function. Conversely for those patients with no residual renal function maintained on dialysis (and hence not at risk of CIN) then CE-CTA can be offered as an alternative to CE-MRA if administration of GBCA is thought high risk. Aside from the preclusion of those with renal impairment, the adverse event rate experienced with low-osmolar iodinated contrast media has been demonstrated to be low in out-patients, with most of these experiencing only mild side effects [3].

From an interpretation perspective heavily calcified arterial disease can be challenging to accurately evaluate on CTA; metal prostheses may produce obscuring artefact and although CE-CTA generally assesses stents well, small-calibre stents remain challenging to analyse. In many institutions, CE-CTA is employed as the initial study of choice for investigation for vascular disease, particularly given the now widespread availability of multislice technology.

## Methods

This study was conducted in a 900-bed teaching hospital providing vascular surgical services as well as a regional renal medicine unit over a 12-year period. The contrast-enhanced MRI angiography (CE-MRA) service was initially introduced in June 1998 after a CE-MRA-capable MRI system was installed—a 1.5-T Philips Gyroscan ACS-NT MRI Scanner (Philips Medical Systems, Best, The Netherlands), including a contrast pump injector (Medrad Spectris, Medrad, Indianola, PA). Following an upgrade in 2002, the scanner was enhanced with a software and table upgrade to allow moving table peripheral angiography plus phased array coils enabling implementation of parallel imaging techniques. A second MRI scanner was added in 2009—a 1.5-T Siemens Magnetom Avanto (Siemens Healthcare, Erlangen, Germany), again with a Medrad contrast pump injector and a dedicated peripheral vascular phased array coil. Several generations of CT scanners have been employed in this time, the single detector row scanner initially installed in 1997 that was effectively unable to perform extended CTA studies being supplemented by a four-detetctor row Siemens Somatom 2 in 2002 upon which lower limb angiographic studies could be performed, although with limited slice resolution. A 64-detector row Toshiba Aquilion CT scanner (Toshiba Medical Systems, Tochigi-ken, Japan) equipped with a Medrad Stellant dual-head contrast pump injector was installed in 2007, replacing the original single-detector-row machine and allowing thin collimation peripheral run-off studies. A second identical 64-slice Toshiba scanner was installed in 2009, bringing the complement of CT scanners to three: one 4 detector row and two 64 detector row.

The baseline angiography and interventional vascular caseload was established for the year June 1997 to the end of May 1998. The subsequent workload to the end of May 2010 was prospectively collated each year and comparison drawn with the CE-MRA and latterly CE-CTA numbers. Latterly CE-CTA numbers were also collected for additional comparison. The numbers of investigations, procedures performed and body area examined (grouped into aortic arch and carotid arteries, thoracic aorta, renal vascular studies, abdominal aorta, aortoiliac and lower limb arterial run-off plus venographic studies) were recorded. CT pulmonary angiograms and more recently CT coronary angiograms have been specifically excluded, with only CT angiographic vascular studies generated via the vascular service evaluated. Ultrasound is not employed for primary investigation of lower limb peripheral vascular disease in our institution (ultrasound is reserved for graft surveillance), and hence has not been studied.

Changes in waiting times over the decade have been compared to baseline, whilst a marginal cost analysis for conventional diagnostic angiographic procedures was made accounting for consumables (contrast media, film, archive media, catheters, guide wires etc.), staffing (radiographers, nursing staff and radiologist time) and hospital bed costs. Marginal cost for CE-CTA and CE-MRA was also calculated taking similar relevant parameters into consideration.

The equivalent radiation dose for CE-CTA performed on our 64-detector-row scanner was estimated by calculating the mean of the effective dose of all such studies performed in the final month of data acquisition [9, 10].

The angiographic, CT and MRI workload was evaluated up to May 2010 using the Radiology Information System (RIS) and compared with the workload in May 1998 using the vascular theatre logs. Alterations in workload pattern have been correlated historically to the changes in the imaging environment at the local and national/international level that have been perceived to have had an impact.

## Results

**Fig. 2:**
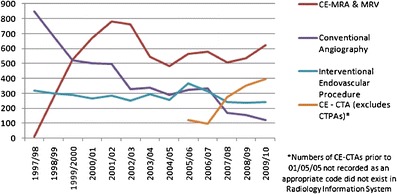
CE-MRA, conventional diagnostic and interventional angiography

The study period, numbering 13 years in total (including baseline years), runs from 1 June 1997 to 31 May 2010, during which time there have been 8,769 invasive angiographic procedures, of which 5,105 were diagnostic vascular and 3,664 were interventional vascular procedures. In the same period there were 6,859 CE-MRA procedures, with the overall number of vascular studies numbering 16,872. There was a year-on-year rise in the number of CE-MRA studies performed each year from 1997/98 (13 studies) to 2002/03 (760 studies) with a reciprocal 50.4 % drop in conventional diagnostic angiography during the same period (847–328 studies, Fig. 2). The number of interventional angiographic procedures remained largely constant. A small drop in the number of CE-MRA studies occurred after 2003/04.

**Fig. 3:**
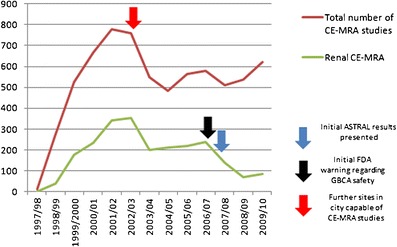
CE-MRA renal studies

Comparison of monthly angiographic workloads at the start and end of data acquisition demonstrated stark differences in practice. In May 1998, 52 conventional diagnostic angiograms were performed, along with 22 invasive interventional procedures. In May 2010, 3 conventional diagnostic angiograms were performed, along with 23 invasive interventional procedures. Meanwhile 40 CE-MRA studies and 23 CE-CTA studies were undertaken in this period. Figure 3 depicts the numbers of CE-MRA studies, with the timing of the FDA warning regarding the use of GBCAs and presentation of initial results from the ASTRAL trial highlighted (see later discussion).

**Fig. 4:**
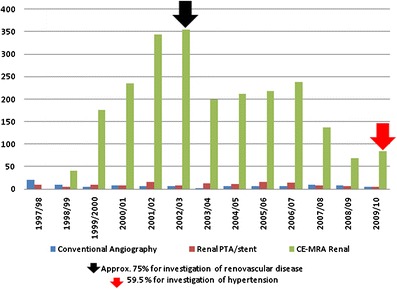
Total numbers of renal investigations

**Fig. 5:**
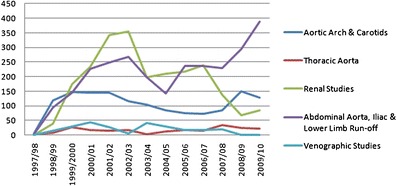
CE-MRA by body site

**Fig. 6:**
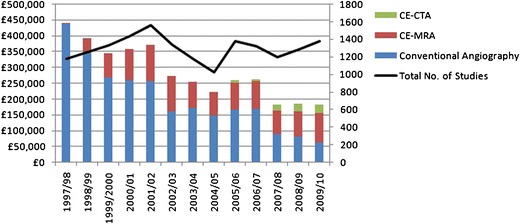
Cost of the diagnostic angiography service compared to total number of studies

The number of renal angiographic investigations performed is detailed in Fig. 4, demonstrating a very rapid initial growth in renal CE-MRA in particular and subsequent decline on a background of relatively constant low numbers of invasive renal angiograms, angioplasties and angioplasty/stent procedures performed. In 2009/10, 50 of the 84 CE-MRAs (59.5 %) performed were for investigation of hypertension (red arrow in Fig. 4). In 2002/03, exact referral patterns are difficult to ascertain because of a change in the Radiology Information System coding, but around 75 % of studies were for investigation of possible renovascular disease (black arrow in Fig. 4). The number of CE-MRA studies by body region is demonstrated in Fig. 5, with the largest increase in studies of the abdominal aorta, iliac arteries and lower limb run-off, with a reciprocal drop in the conventional invasive angiograms performed for this purpose. Until 2003, the numbers of total radiological vascular investigations and procedures showed a steady rise, and have since remained roughly constant to the present day (Fig. 6).

Waiting times have varied over this period, with the wait in 1997 for a non-urgent conventional invasive angiogram for investigation of claudication peaking at 7 months. This had reduced to 3 weeks by 2003 and stands at a maximum of 3 weeks currently. The waiting period for CE-MRA has varied with popularity and availability of the technique, rising to around 4 months in 2003 following the service’s inception and prior to the second scanner acquisition. This has also reduced to less than a 3-week wait currently, with CE-CTA requiring a similar waiting time for non-urgent out-patient studies.

Marginal cost analyses were performed in 2001 and 2009, with costs for CE-MRA actually reducing from £149 (€185) per study to around £100 (€125) currently. Conventional diagnostic angiography, including the cost of a day or overnight bed, cost £515 (€640) in 2001, and this was similar in 2009. The CE-CTA marginal cost was estimated at £68 (€85) per study in 2009. The overall cost of the diagnostic angiography service is demonstrated in Fig. 6, with the individual costs of conventional diagnostic angiography, CE-MRA and CE-CTA included.

The mean effective dose for a selection of peripheral angiograms was performed using the dose-length product [11], with a mean dose of 16.6mSv utilising an ICRP 60-tissue weighting factor for body CT [12, 13].

## Discussion

The advent of non-invasive vascular imaging using CE-MRA and CE-CTA has resulted in non-invasive alternatives to invasive diagnostic angiography providing excellent quality three-dimensional images whilst avoiding the potential complications associated with invasive procedures. Both CE-MRA and CE-CTA are superior to ultrasound in their ability to image the entirety of the pertinent vascular tree, avoiding compromise of image quality by depth in the body or interposed bowel gas. Another major advantage over ultrasound is the depiction of the vascular tree in a familiar angiographic format with very low inter-observer variability enhancing clinical credibility and allowing straightforward discussion in the multidisciplinary team environment. The overall impact of CE-MRA and CE-CTA in our institution has been a marked reduction in the conventional diagnostic angiography workload, with extensive use of CE-MRA and more recently CE-CTA in a wide range of clinical scenarios, with tailoring of modality to individual cases bearing in mind the ALARA principle. The volume and choice of the type of examinations performed have been influenced by modality availability, clinical trends and technical developments, as well as consequential theoretical and real adverse effects. The impact has varied over time in relation to various factors as per the following discussions.

### Renovascular imaging

A steady increase in diagnostic vascular imaging activity was apparent up until 2002/03, with an additional 382 patients (32.4 %) examined overall in the year 2001/02 as compared to 1997/98. This is in part due to a proliferation in renovascular imaging in this period. Whilst only 22 conventional renal angiograms were performed in the year prior to the advent of CE-MRA in 1997/98 for investigation of suspected renal artery stenosis, in 2002/03 377 renovascular studies were undertaken, accounting for almost all the increase in diagnostic vascular imaging. The majority were for investigation of possible atherosclerotic renovascular disease as a cause of renal impairment with or without hypertension, mainly from the renal medicine service and cardiology. Indeed referrals also came from renal physicians and cardiologists based at other institutions in and around the city. This was due to the non-invasive nature, good sensitivity and specificity relative to digital subtraction angiography, and great superiority of CE-MRA compared to Doppler ultrasound [14]. These factors dramatically reduced the threshold for investigation of suspected renovascular disease. A reduction in the total number of renal diagnostic procedures performed at our site in 2002/03 is accounted for partly by a change in service provision with another city site starting a CE-MRA service. However, another factor is that a large backlog of chronic kidney disease patients attending clinics hitherto not investigated had by then been scanned for suspected renovascular disease, with largely only new presentations being referred for CE-MRA subsequently. The number of renal revascularisations remained consistently low reflecting a local scepticism among the renal physicians regarding the value of revascularisation although they were clearly keen to document the nature of disease in their patients. This situation is likely to continue given the efficacy of modern medical management and slower rate of renal function decline than previously believed. Renal vascular intervention in our institution is now largely reserved for patients with recurrent ‘flash’ pulmonary oedema, rapid renal functional decline or uncontrolled malignant hypertension despite maximal medical therapy, although the new technique of renal denervation may reverse this [15, 16]. Development of another CE-MRA capable site also contributed to the drop in total number of studies at our institution in 2002/03 (red arrow in Fig. 3). The Angioplasty and Stent for Renal Artery Lesions trial (ASTRAL) in which our centre participated has been by far the largest trial in atherosclerotic renovascular disease (ARVD), recruiting 806 patients [15]. The trial was designed to address whether renal revascularisation with balloon angioplasty/stent could prevent progressive renal failure among ARVD patients. The trial found substantial risks but no evidence of a worthwhile clinical benefit from revascularisation in patients with ARVD. However, the effect of the dissemination of the results of the ASTRAL trial was actually minimal at our institution (blue arrow in Fig. 3), largely because of local renal physician scepticism and the prior significant reduction in imaging of patients with renal impairment as a result of newly recognised GBCA toxicity in patients with severe renal failure, a phenomenon discussed below [17].

### Nephrogenic systemic fibrosis

A decrease in the total number of CE-MRA studies was observed in 2007/08, reducing from 580 to 509 studies (a 12.2 % drop). This was largely due to the large relative reduction in renal studies in this period, demonstrated in Fig. 5, attributable to the initial reporting in May 2006 of an association between the use of GBCAs and the development of nephrogenic systemic fibrosis (NSF). NSF is a serious disorder characterised by hardening and thickening of the skin and body tissues. Initially described as ‘*scleromyxoedema-like skin thickening*’ affecting the limbs and trunk [18], it was solely seen in dialysis patients and was first titled nephrogenic fibrosing dermopathy [19], subsequently renamed nephrogenic systemic fibrosis. Reviews noted the universal setting of renal failure but not the cause [20–22]. Prior to this there had been no concerns regarding the use of GBCA in haemodialysis patients, with prompt excretory rates at haemodialysis following GBCA administration and initial research suggesting GBCAs in the doses usually employed had an excellent safety profile and were less nephrotoxic than iodinated contrast [5, 23]. This led to widespread use of CE-MRA in patients with impaired renal function where examinations employing iodine-based contrast media engender significant risk. In 2006, GBCAs were first proposed as a potential factor in the development of NSF [5], and research indicated an association with the recent use of GBCAs, particularly in high dose on a background of chronic dialysis-dependent renal failure (or less commonly acute renal failure) [24]. Further evidence was the discovery of traces of gadolinium in tissue biopsies from affected patients [25, 26]. A United States Food and Drug Administration warning regarding the use of GBCAs was released in June 2006 [27]. Currently, the linear chelate compounds (gadopentetate dimeglumine, gadodiamide and gadoversetamide) are contra-indicated in those patients with a glomerular filtration rate (GFR) of less than 30 ml/min and avoiding GBCA use with other compounds is recommended unless absolutely clinically necessary and then with minimum dose. This has led to a change in clinical practise in order to identify those at risk such as those with diabetes, hypertension or on nephrotoxic medication in whom knowledge of GFR is important prior to decision making on imaging mode [28]. Recent reviews indicate that the risk of NSF can be eliminated by careful management of risk factors, with up to a ten-fold risk reduction by elimination of just one risk factor [29]. This reduction in the applicability of CE-MRA has spurred the development of newer non-enhanced MRA techniques, such as arterial spin labelling for aortic or renal imaging, as well as ECG-gated 3D partial-Fourier fast spin echo sequences in the evaluation of peripheral arterial disease [30, 31] for those patients with end-stage renal disease.

### Role of CT angiography

**Fig. 7:**
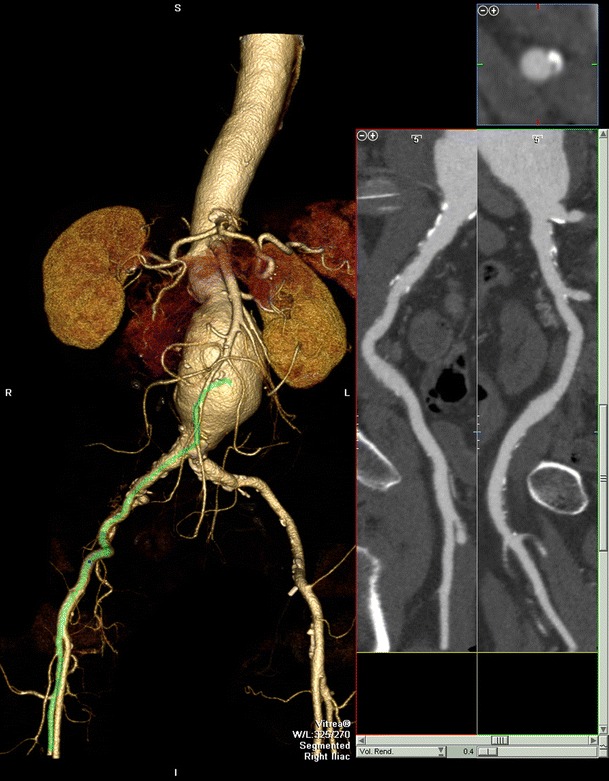
Three-dimensional volume-rendered image and curve planar reformat (CPR) along vessel centreline for right iliac arteries from CTA performed for assessment of abdominal aortic aneurysm to assess potential for endovascular repair. Infrarenal aneurysm terminates at the aortic bifurcation and CPR (delineated by green line on VR image) demonstrates satisfactory calibre right iliac access without stenotic disease

The increase in the use of CE-CTA over the final 5 years of the study period merits discussion, with an increase in CE-CTA (excluding CT pulmonary angiography and CT coronary angiography) from 122 studies in 2005/06 to 396 studies in the final year of data collection. This is a technology-driven phenomenon with utilisation having been revolutionised by multidetector row scanners with 16 or more detector rows. A wide range of clinical applications are described, including traumatic injuries, embolic phenomena, aneurysms and atherosclerotic disease [32]. Figure 7 demonstrates a clinical application of CE-CTA. Good accuracy has been demonstrated with 4- and 16-slice scanners, but it has particularly been with the advent of ‘64-slice’ scanners that CE-CTA has become a more mature and robust modality. In the lower extremities accuracy has been shown to be good for claudicant patients although its role in critical lower limb ischaemia remains relatively controversial and unproven [8, 33]. CE-CTA can play a useful role in those patients established on renal dialysis with no residual renal function, as there is no concern regarding potential contrast-induced nephropathy although potential adverse effects on the myocardium and volume loading remain, whilst difficulties in interpretation are encountered due to heavily calcification significantly obscuring the lumen, particularly in small vessels. This calcification is of itself useful information to the surgeon as regards the quality of the vessels for clamping and anastomosis, although there is anecdotal evidence that it may unnecessarily dissuade surgeons from operating and blooming artefacts can certainly make calcified plaques appear much more pronounced than in physical reality. CE-CTA certainly offers improved visualisation of metallic stents and stent grafts and their lumens compared to CE-MRA though evaluation of very small calibre stents remains problematic. CE-CTA is preferred in patients in whom CE-MRA is contra-indicated, such as those with pacemakers, other metallic implants or claustrophobia. Despite the requirement for administration of iodine-based contrast media and exposure to ionising radiation [34], patients experience fewer adverse events than from either contrast angiography or CE-MRA [35]. The risk of contrast-induced nephropathy can be reduced by prior volume expansion with saline or sodium bicarbonate, and careful monitoring of Metformin use [4]. Though a lesser radiation burden than digital subtraction angiography (DSA), CE-CTA is a relatively high-dose procedure, on average 13.7 mSv per study using a 4-slice scanner [36, 37]. This compares with a value of around 12 mSv on average for conventional abdominal angiography with DSA, although this figure varies between 4 and 48 mSv depending on the difficulty of the procedure and operator expertise [38, 39]. The CT angiography dose is higher with more modern 64-slice scanners, and analysis of our own examinations indicates an average dose of 16.6 mSv per study. However, it should be noted that this is based on ICRP 60 [12]. There is no currently accepted conversion factor for peripheral angiograms and the use of ICRP 103 recommendations would likely significantly increase this. A number of dose reduction strategies are available, such as tube current modulation and alteration of the tube current and voltage dependant on the patient’s body mass index [40] and more recently iterative reconstruction techniques [41]. Lack of information on flow dynamics is a major drawback of CT in comparison to both CE-MRA and DSA.

### Overall diagnostic demand

Interestingly, despite large variations in angiographic modality choice and availability over a decade, then bar the renal angiographic threshold being reduced, the overall number of diagnostic investigations through the vascular surgical service has remained largely constant, now with a total number of studies usually between 1,200 and 1,400 per year. The only significant departure from this, 1,014 studies performed in 2004/05, was a result of transfer of a proportion of CE-MRA services to two further city sites within the health board, which commenced a CE-MRA service. This suggests that demand for services has not significantly altered, but evolution of technology and its availability has necessitated change.

### Impact on interventional services

The significant shift to non-invasive vascular imaging is well demonstrated by comparison of monthly diagnostic workloads at the start and end of data acquisition, with minimal numbers of invasive conventional angiography now performed, largely replaced by non-invasive CE-MRA and CE-CTA. Although the number of interventional vascular cases has remained broadly constant over the study period, there has been a significant trend to an increase in the complexity of time-consuming cases, such as endovascular aneurysm repair (EVAR). Meanwhile there has been a reduction in more straightforward angioplasty (although a lesser role than previously thought in claudication due to femoropopliteal disease) and thrombolysis. Despite the considerable increase in the time required in the interventional suite to perform procedures such as EVAR, the uptake of non-invasive angiography techniques has helped free up the time in the vascular radiology theatre suite previously reserved for routine diagnostic angiography, allowing the more extensive access required for complex interventional procedures. The relatively constant number of interventional procedures is likely a combination of the reduction in infra-inguinal angioplasties for claudication in line with local practice and the reduction in thrombolysis.

### Cost analysis and impact on departmental workflow

Figure 6 depicts the trend of generally constant diagnostic activity and constantly reducing cost, demonstrating the cost-effectiveness of these imaging techniques. Coupled with their non-invasive nature, the benefit of performing the majority of diagnostic vascular imaging by CE-MRA or CE-CTA is clear. This cost analysis does not include the initial outlay on equipment, with our department now hosting two CE-MRA capable units and three multi-slice CT scanners in line with the general demand for these modalities, which represents a significant investment. Cost analysis, though complex, plays an important role in determining future departmental imaging strategies, allowing projections of costs in order to help plan future provisions for imaging whilst enabling analysis of the impact of previous changes in use of imaging modalities and referral patterns, as demonstrated by our data. Planning for any future structural changes in a department should be undertaken, for instance leaving space for additional scanners in new buildings or earmarking obsolete or unused areas for a change in use. Waiting times also impact significantly on a department’s workflow and interaction with other departments, and consideration of extended working hours, clear indications for patient referral, the need for additional scanners and general measures such as sequence rationalisation in MRI all play a part in reducing this as much as possible. Invasive diagnostic angiography conventionally required at least a day bed or even overnight stay, adding additional strain in terms of resources along with adequate nursing and medical cover. CE-CTA and CE-MRA have removed this requirement, with procedures performed as an out-patient with no need for an extended hospital stay and a vastly reduced incidence of post-procedural complications.

### Educational impact

The potential for marked changes in diagnostic practice is demonstrated in Fig. 2. One radiology educational consequence has been that the drop in diagnostic angiography has led to more limited opportunities to acquire basic vascular skills although this is a small change compared to other alterations in interventional vascular radiology training. Continuing medical education plays a crucial role in ensuring radiologists keep apace with this, both through formal learning or teaching but also via self-directed learning. Trainee radiologists should be aware of the potential for such alterations in practice and the requirement to be flexible throughout their career, underlining the need for good basic training in all modalities and continuing medical education. By keeping abreast of current publications and guidelines and maintaining good links with the reporting clinicians, established radiologists will be better placed to anticipate changes in modality demand and develop approaches to ensure the standard of care is maintained.

## Conclusion

Vascular imaging has undergone a significant metamorphosis in little over a decade, with a major decline in conventional diagnostic angiography and a corresponding boom in CE-MRA and more recently CE-CTA. The modalities each have their strengths and weaknesses but they have become firmly established and we have demonstrated cost-effective, time-effective and safe diagnostic angiographic use.
